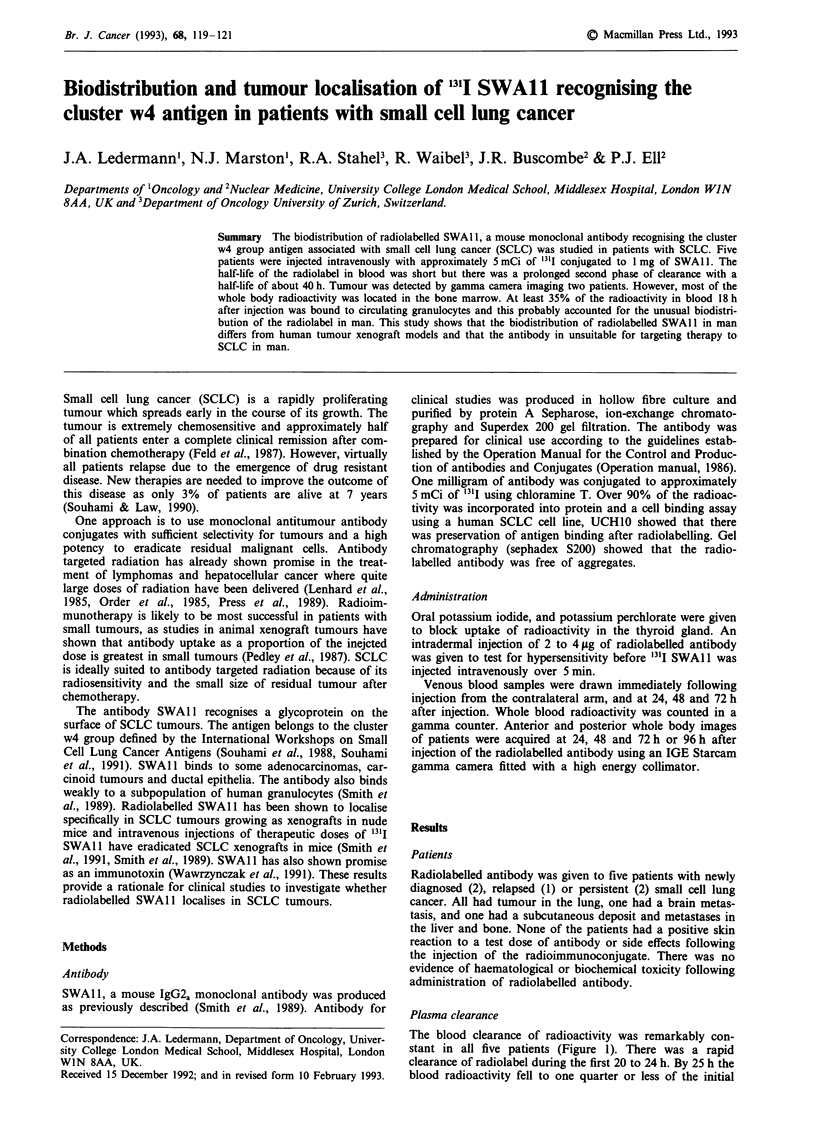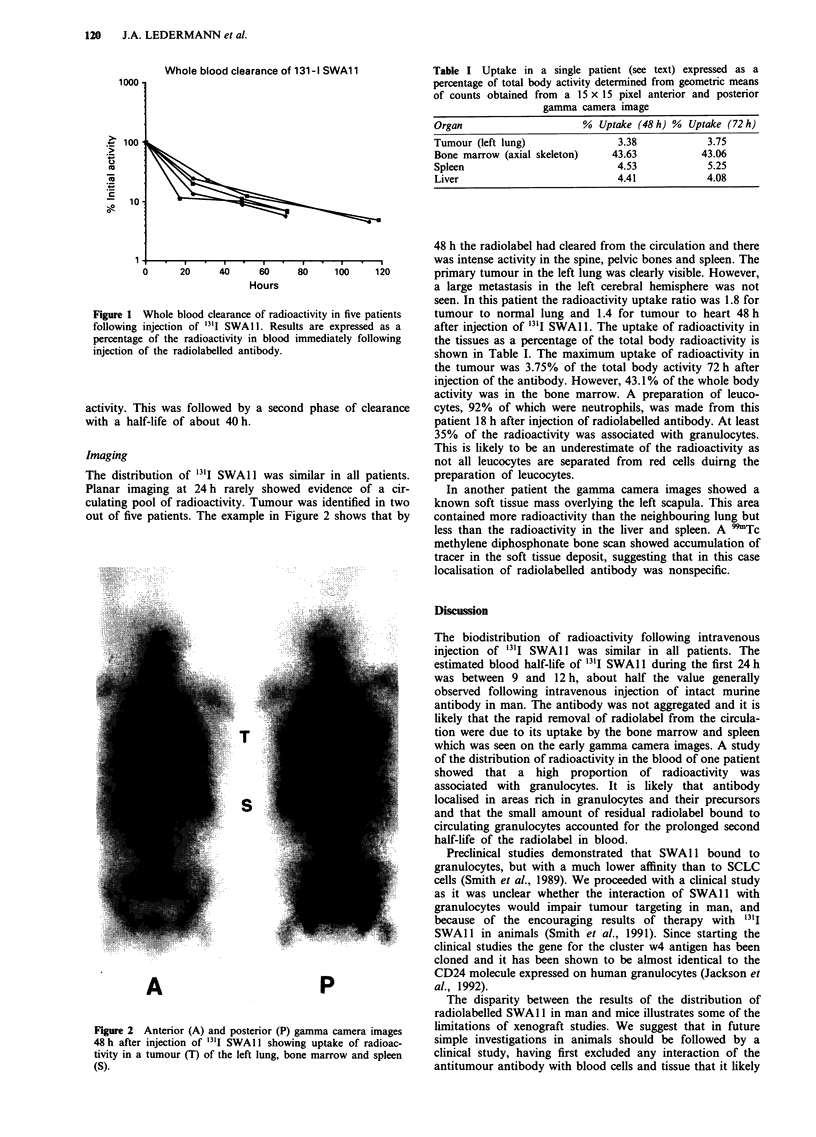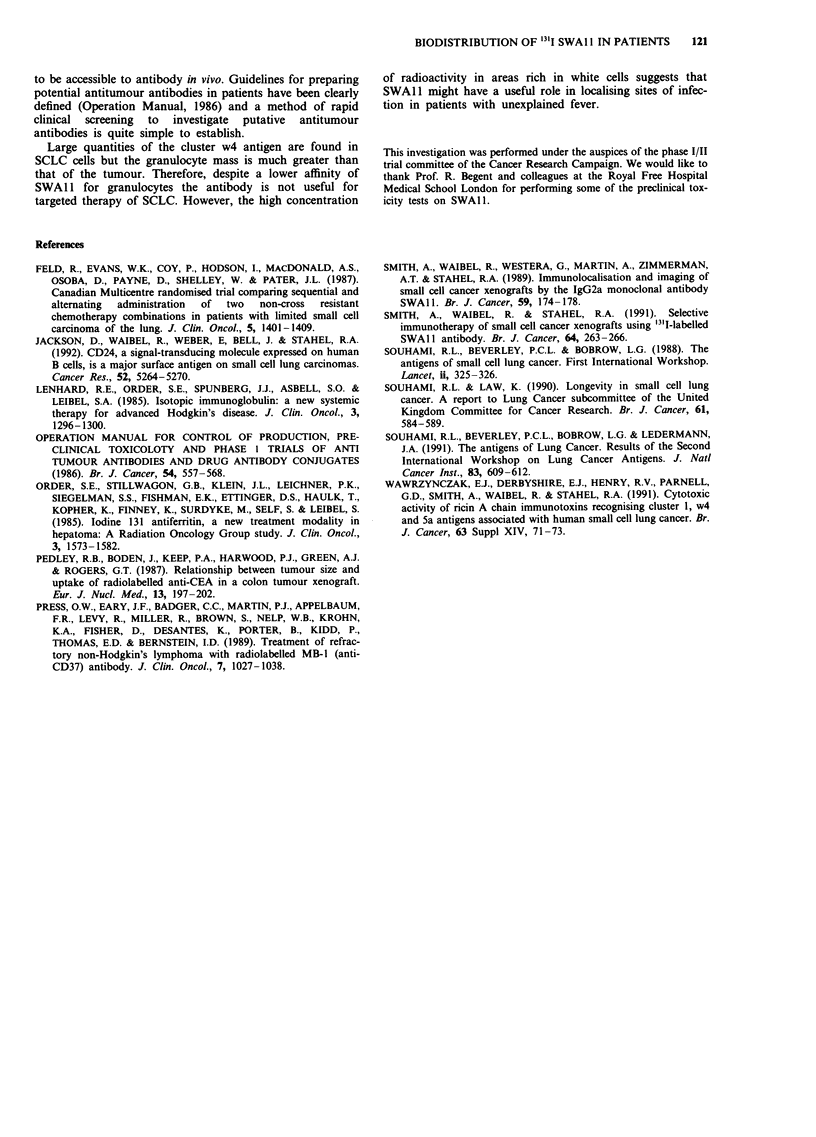# Biodistribution and tumour localisation of 131I SWA11 recognising the cluster w4 antigen in patients with small cell lung cancer.

**DOI:** 10.1038/bjc.1993.297

**Published:** 1993-07

**Authors:** J. A. Ledermann, N. J. Marston, R. A. Stahel, R. Waibel, J. R. Buscombe, P. J. Ell

**Affiliations:** Department of Oncology, University College London Medical School, Middlesex Hospital, UK.

## Abstract

**Images:**


*Br. J. Cancer* (1993), **68**, 119–121 © Macmillan Press Ltd., 1993

### Biodistribution and tumour localisation of $^{131}$I SWA11 recognising the cluster w4 antigen in patients with small cell lung cancer

J.A. Ledermann[1], N.J. Marston[1], R.A. Stahel[3], R. Waibel[3], J.R. Buscombe[2] & P.J. Ell[2]

*Departments of [1]Oncology and [2]Nuclear Medicine, University College London Medical School, Middlesex Hospital, London W1N 8AA, UK and [3]Department of Oncology University of Zurich, Switzerland.*

**Summary** The biodistribution of radiolabelled SWA11, a mouse monoclonal antibody recognising the cluster w4 group antigen associated with small cell lung cancer (SCLC) was studied in patients with SCLC. Five patients were injected intravenously with approximately 5 mCi of $^{131}$I conjugated to 1 mg of SWA11. The half-life of the radiolabel in blood was short but there was a prolonged second phase of clearance with a half-life of about 40 h. Tumour was detected by gamma camera imaging two patients. However, most of the whole body radioactivity was located in the bone marrow. At least 35% of the radioactivity in blood 18 h after injection was bound to circulating granulocytes and this probably accounted for the unusual biodistribution of the radiolabel in man. This study shows that the biodistribution of radiolabelled SWA11 in man differs from human tumour xenograft models and that the antibody in unsuitable for targeting therapy to SCLC in man.

Small cell lung cancer (SCLC) is a rapidly proliferating tumour which spreads early in the course of its growth. The tumour is extremely chemosensitive and approximately half of all patients enter a complete clinical remission after combination chemotherapy (Feld *et al.*, 1987). However, virtually all patients relapse due to the emergence of drug resistant disease. New therapies are needed to improve the outcome of this disease as only 3% of patients are alive at 7 years (Souhami & Law, 1990).

One approach is to use monoclonal antitumour antibody conjugates with sufficient selectivity for tumours and a high potency to eradicate residual malignant cells. Antibody targeted radiation has already shown promise in the treatment of lymphomas and hepatocellular cancer where quite large doses of radiation have been delivered (Lenhard *et al.*, 1985, Order *et al.*, 1985, Press *et al.*, 1989). Radioimmunotherapy is likely to be most successful in patients with small tumours, as studies in animal xenograft tumours have shown that antibody uptake as a proportion of the inejcted dose is greatest in small tumours (Pedley *et al.*, 1987). SCLC is ideally suited to antibody targeted radiation because of its radiosensitivity and the small size of residual tumour after chemotherapy.

The antibody SWA11 recognises a glycoprotein on the surface of SCLC tumours. The antigen belongs to the cluster w4 group defined by the International Workshops on Small Cell Lung Cancer Antigens (Souhami *et al.*, 1988, Souhami *et al.*, 1991). SWA11 binds to some adenocarcinomas, carcinoid tumours and ductal epithelia. The antibody also binds weakly to a subpopulation of human granulocytes (Smith *et al.*, 1989). Radiolabelled SWA11 has been shown to localise specifically in SCLC tumours growing as xenografts in nude mice and intravenous injections of therapeutic doses of $^{131}$I SWA11 have eradicated SCLC xenografts in mice (Smith *et al.*, 1991, Smith *et al.*, 1989). SWA11 has also shown promise as an immunotoxin (Wawrzynczak *et al.*, 1991). These results provide a rationale for clinical studies to investigate whether radiolabelled SWA11 localises in SCLC tumours.

### Methods

### *Antibody*

SWA11, a mouse $IgG2_a$ monoclonal antibody was produced as previously described (Smith *et al.*, 1989). Antibody for clinical studies was produced in hollow fibre culture and purified by protein A Sepharose, ion-exchange chromatography and Superdex 200 gel filtration. The antibody was prepared for clinical use according to the guidelines established by the Operation Manual for the Control and Production of antibodies and Conjugates (Operation manual, 1986). One milligram of antibody was conjugated to approximately 5 mCi of $^{131}$I using chloramine T. Over 90% of the radioactivity was incorporated into protein and a cell binding assay using a human SCLC cell line, UCH10 showed that there was preservation of antigen binding after radiolabelling. Gel chromatography (sephadex S200) showed that the radiolabelled antibody was free of aggregates.

### *Administration*

Oral potassium iodide, and potassium perchlorate were given to block uptake of radioactivity in the thyroid gland. An intradermal injection of 2 to 4 $\mu$g of radiolabelled antibody was given to test for hypersensitivity before $^{131}$I SWA11 was injected intravenously over 5 min.

Venous blood samples were drawn immediately following injection from the contralateral arm, and at 24, 48 and 72 h after injection. Whole blood radioactivity was counted in a gamma counter. Anterior and posterior whole body images of patients were acquired at 24, 48 and 72 h or 96 h after injection of the radiolabelled antibody using an IGE Starcam gamma camera fitted with a high energy collimator.

### Results

### *Patients*

Radiolabelled antibody was given to five patients with newly diagnosed (2), relapsed (1) or persistent (2) small cell lung cancer. All had tumour in the lung, one had a brain metastasis, and one had a subcutaneous deposit and metastases in the liver and bone. None of the patients had a positive skin reaction to a test dose of antibody or side effects following the injection of the radioimmunoconjugate. There was no evidence of haematological or biochemical toxicity following administration of radiolabelled antibody.

### *Plasma clearance*

The blood clearance of radioactivity was remarkably constant in all five patients (Figure 1). There was a rapid clearance of radiolabel during the first 20 to 24 h. By 25 h the blood radioactivity fell to one quarter or less of the initial

Correspondence: J.A. Ledermann, Department of Oncology, University College London Medical School, Middlesex Hospital, London W1N 8AA, UK.
Received 15 December 1992; and in revised form 10 February 1993.

**Figure 1** Whole blood clearance of radioactivity in five patients following injection of $^{131}I$ SWA11. Results are expressed as a percentage of the radioactivity in blood immediately following injection of the radiolabelled antibody.

activity. This was followed by a second phase of clearance with a half-life of about 40 h.

*Imaging*

The distribution of $^{131}I$ SWA11 was similar in all patients. Planar imaging at 24 h rarely showed evidence of a circulating pool of radioactivity. Tumour was identified in two out of five patients. The example in Figure 2 shows that by

**Figure 2** Anterior (A) and posterior (P) gamma camera images 48 h after injection of $^{131}I$ SWA11 showing uptake of radioactivity in a tumour (T) of the left lung, bone marrow and spleen (S).

**Table I** Uptake in a single patient (see text) expressed as a percentage of total body activity determined from geometric means of counts obtained from a 15 × 15 pixel anterior and posterior gamma camera image

| *Organ* | *% Uptake (48 h)* | *% Uptake (72 h)* |
|---|---|---|
| Tumour (left lung) | 3.38 | 3.75 |
| Bone marrow (axial skeleton) | 43.63 | 43.06 |
| Spleen | 4.53 | 5.25 |
| Liver | 4.41 | 4.08 |

48 h the radiolabel had cleared from the circulation and there was intense activity in the spine, pelvic bones and spleen. The primary tumour in the left lung was clearly visible. However, a large metastasis in the left cerebral hemisphere was not seen. In this patient the radioactivity uptake ratio was 1.8 for tumour to normal lung and 1.4 for tumour to heart 48 h after injection of $^{131}I$ SWA11. The uptake of radioactivity in the tissues as a percentage of the total body radioactivity is shown in Table I. The maximum uptake of radioactivity in the tumour was 3.75% of the total body activity 72 h after injection of the antibody. However, 43.1% of the whole body activity was in the bone marrow. A preparation of leucocytes, 92% of which were neutrophils, was made from this patient 18 h after injection of radiolabelled antibody. At least 35% of the radioactivity was associated with granulocytes. This is likely to be an underestimate of the radioactivity as not all leucocytes are separated from red cells duirng the preparation of leucocytes.

In another patient the gamma camera images showed a known soft tissue mass overlying the left scapula. This area contained more radioactivity than the neighbouring lung but less than the radioactivity in the liver and spleen. A $^{99m}Tc$ methylene diphosphonate bone scan showed accumulation of tracer in the soft tissue deposit, suggesting that in this case localisation of radiolabelled antibody was nonspecific.

### Discussion

The biodistribution of radioactivity following intravenous injection of $^{131}I$ SWA11 was similar in all patients. The estimated blood half-life of $^{131}I$ SWA11 during the first 24 h was between 9 and 12 h, about half the value generally observed following intravenous injection of intact murine antibody in man. The antibody was not aggregated and it is likely that the rapid removal of radiolabel from the circulation were due to its uptake by the bone marrow and spleen which was seen on the early gamma camera images. A study of the distribution of radioactivity in the blood of one patient showed that a high proportion of radioactivity was associated with granulocytes. It is likely that antibody localised in areas rich in granulocytes and their precursors and that the small amount of residual radiolabel bound to circulating granulocytes accounted for the prolonged second half-life of the radiolabel in blood.

Preclinical studies demonstrated that SWA11 bound to granulocytes, but with a much lower affinity than to SCLC cells (Smith *et al.*, 1989). We proceeded with a clinical study as it was unclear whether the interaction of SWA11 with granulocytes would impair tumour targeting in man, and because of the encouraging results of therapy with $^{131}I$ SWA11 in animals (Smith *et al.*, 1991). Since starting the clinical studies the gene for the cluster w4 antigen has been cloned and it has been shown to be almost identical to the CD24 molecule expressed on human granulocytes (Jackson *et al.*, 1992).

The disparity between the results of the distribution of radiolabelled SWA11 in man and mice illustrates some of the limitations of xenograft studies. We suggest that in future simple investigations in animals should be followed by a clinical study, having first excluded any interaction of the antitumour antibody with blood cells and tissue that it likely to be accessible to antibody *in vivo*. Guidelines for preparing potential antitumour antibodies in patients have been clearly defined (Operation Manual, 1986) and a method of rapid clinical screening to investigate putative antitumour antibodies is quite simple to establish.

Large quantities of the cluster w4 antigen are found in SCLC cells but the granulocyte mass is much greater than that of the tumour. Therefore, despite a lower affinity of SWA11 for granulocytes the antibody is not useful for targeted therapy of SCLC. However, the high concentration of radioactivity in areas rich in white cells suggests that SWA11 might have a useful role in localising sites of infection in patients with unexplained fever.

This investigation was performed under the auspices of the phase I/II trial committee of the Cancer Research Campaign. We would like to thank Prof. R. Begent and colleagues at the Royal Free Hospital Medical School London for performing some of the preclinical toxicity tests on SWA11.

### References

FELD, R., EVANS, W.K., COY, P., HODSON, I., MACDONALD, A.S., OSOBA, D., PAYNE, D., SHELLEY, W. & PATER, J.L. (1987). Canadian Multicentre randomised trial comparing sequential and alternating administration of two non-cross resistant chemotherapy combinations in patients with limited small cell carcinoma of the lung. *J. Clin. Oncol.*, **5**, 1401–1409.

JACKSON, D., WAIBEL, R., WEBER, E, BELL, J. & STAHEL, R.A. (1992). CD24, a signal-transducing molecule expressed on human B cells, is a major surface antigen on small cell lung carcinomas. *Cancer Res.*, **52**, 5264–5270.

LENHARD, R.E., ORDER, S.E., SPUNBERG, J.J., ASBELL, S.O. & LEIBEL, S.A. (1985). Isotopic immunoglobulin: a new systemic therapy for advanced Hodgkin's disease. *J. Clin. Oncol.*, **3**, 1296–1300.

OPERATION MANUAL FOR CONTROL OF PRODUCTION, PRECLINICAL TOXICOLOTY AND PHASE 1 TRIALS OF ANTI TUMOUR ANTIBODIES AND DRUG ANTIBODY CONJUGATES (1986). *Br. J. Cancer*, **54**, 557–568.

ORDER, S.E., STILLWAGON, G.B., KLEIN, J.L., LEICHNER, P.K., SIEGELMAN, S.S., FISHMAN, E.K., ETTINGER, D.S., HAULK, T., KOPHER, K., FINNEY, K., SURDYKE, M., SELF, S. & LEIBEL, S. (1985). Iodine 131 antiferritin, a new treatment modality in hepatoma: A Radiation Oncology Group study. *J. Clin. Oncol.*, **3**, 1573–1582.

PEDLEY, R.B., BODEN, J., KEEP, P.A., HARWOOD, P.J., GREEN, A.J. & ROGERS, G.T. (1987). Relationship between tumour size and uptake of radiolabelled anti-CEA in a colon tumour xenograft. *Eur. J. Nucl. Med.*, **13**, 197–202.

PRESS, O.W., EARY, J.F., BADGER, C.C., MARTIN, P.J., APPELBAUM, F.R., LEVY, R., MILLER, R., BROWN, S., NELP, W.B., KROHN, K.A., FISHER, D., DESANTES, K., PORTER, B., KIDD, P., THOMAS, E.D. & BERNSTEIN, I.D. (1989). Treatment of refractory non-Hodgkin's lymphoma with radiolabelled MB-1 (anti-CD37) antibody. *J. Clin. Oncol.*, **7**, 1027–1038.

SMITH, A., WAIBEL, R., WESTERA, G., MARTIN, A., ZIMMERMAN, A.T. & STAHEL, R.A. (1989). Immunolocalisation and imaging of small cell cancer xenografts by the IgG2a monoclonal antibody SWA11. *Br. J. Cancer*, **59**, 174–178.

SMITH, A., WAIBEL, R. & STAHEL, R.A. (1991). Selective immunotherapy of small cell cancer xenografts using $^{131}$I-labelled SWA11 antibody. *Br. J. Cancer*, **64**, 263–266.

SOUHAMI, R.L., BEVERLEY, P.C.L. & BOBROW, L.G. (1988). The antigens of small cell lung cancer. First International Workshop. *Lancet*, **ii**, 325–326.

SOUHAMI, R.L. & LAW, K. (1990). Longevity in small cell lung cancer. A report to Lung Cancer subcommittee of the United Kingdom Committee for Cancer Research. *Br. J. Cancer*, **61**, 584–589.

SOUHAMI, R.L., BEVERLEY, P.C.L., BOBROW, L.G. & LEDERMANN, J.A. (1991). The antigens of Lung Cancer. Results of the Second International Workshop on Lung Cancer Antigens. *J. Natl Cancer Inst.*, **83**, 609–612.

WAWRZYNCZAK, E.J., DERBYSHIRE, E.J., HENRY, R.V., PARNELL, G.D., SMITH, A., WAIBEL, R. & STAHEL, R.A. (1991). Cytotoxic activity of ricin A chain immunotoxins recognising cluster 1, w4 and 5a antigens associated with human small cell lung cancer. *Br. J. Cancer*, **63** Suppl XIV, 71–73.